# A Systems Approach to Improving Rural Care in Ethiopia

**DOI:** 10.1371/journal.pone.0035042

**Published:** 2012-04-25

**Authors:** Elizabeth H. Bradley, Patrick Byam, Rachelle Alpern, Jennifer W. Thompson, Abraham Zerihun, Yigeremu Abeb, Leslie A. Curry

**Affiliations:** 1 Yale School of Public Health, Global Health Leadership Institute (GHLI), Yale University, New Haven, Connecticut, United States of America; 2 Ethiopian Millennium Rural Initiative (EMRI), Clinton Health Access Initiative (CHAI), Addis Ababa, Ethiopia; Tulane University School of Public Health and Tropical Medicine, United States of America

## Abstract

**Background:**

Multiple interventions have been launched to improve the quality, access, and utilization of primary health care in rural, low-income settings; however, the success of these interventions varies substantially, even within single studies where the measured impact of interventions differs across sites, centers, and regions. Accordingly, we sought to examine the variation in impact of a health systems strengthening intervention and understand factors that might explain the variation in impact across primary health care units.

**Methodology/Principal Findings:**

We conducted a mixed methods positive deviance study of 20 Primary Health Care Units (PHCUs) in rural Ethiopia. Using longitudinal data from the Ethiopia Millennium Rural Initiative (EMRI), we identified PHCUs with consistently higher performance (n = 2), most improved performance (n = 3), or consistently lower performance (n = 2) in the provision of antenatal care, HIV testing in antenatal care, and skilled birth attendance rates. Using data from site visits and in-depth interviews (n = 51), we applied the constant comparative method of qualitative data analysis to identify key themes that distinguished PHCUs with different performance trajectories. Key themes that distinguished PHCUs were 1) managerial problem solving capacity, 2) relationship with the *woreda* (district) health office, and 3) community engagement. In higher performing PHCUs and those with the greatest improvement after the EMRI intervention, health center and health post staff were more able to solve day-to-day problems, staff had better relationships with the *woreda* health official, and PHCU communities' leadership, particularly religious leadership, were strongly engaged with the health improvement effort. Distance from the nearest city, quality of roads and transportation, and cultural norms did not differ substantially among PHCUs.

**Conclusions/Significance:**

Effective health strengthening efforts may require intensive development of managerial problem solving skills, strong relationships with government offices that oversee front-line providers, and committed community leadership to succeed.

## Introduction

Improving access to and quality of rural primary health care, especially in low-income settings, is a global priority [1]–[2]. Multiple efforts have been directed at improving access and use of primary care services, particularly among women and children in rural settings [3]–[5]; however, the success of these interventions varies substantially, even within single studies where the measured impact of interventions can differ across different sites, centers, and regions [6].

Several previous health systems strengthening efforts directed at improving rural primary care in low-income countries have been relatively successful [7]–[9]. At the same time, other efforts have met with weaker outcomes [10]–[12]. Prior research has identified the cost, level and quality of available health services [7], [13]–[14], distance and transportation [14]–[16], and social and cultural norms [17]–[18] as site-level factors influencing the utilization of primary health care services. Although this literature is useful, studies typically report overall effects, limiting our understanding of the heterogeneity in effects across different intervention sites even within the same study. As a result, we know relatively little about why the same set of interventions may be effective in one rural health care unit and less so in another.

Accordingly, we sought to generate hypotheses about factors that may explain the variation in performance across primary health care units. Using longitudinal data from the Ethiopian Millennium Rural Initiative (EMRI), a health systems strengthening effort designed to improve the performance of rural primary health care units (PHCUs) located throughout 4 regions in Ethiopia, we categorized PHCUs into three types: PHCUs with consistently higher performance, PHCUs with the most improved performance, and PHCUs with consistently lower performance. Because our goal was to generate hypotheses and because the literature is limited in this area, we used qualitative methods to explore in rich detail differences among the higher performing, most improved, and lower performing PHCUs. Evidence from this study might be useful in the design and implementation of effective rural primary care strengthening efforts in low-income settings.

## Methods

### Ethics Statement

All research procedures were approved by the Institutional Review Board at the Yale School of Medicine and the Ethiopian Federal Ministry of Health.

### Setting and intervention

Ethiopia, a country of approximately 80 million people, is ranked 174 out of 187 on the Human Development Index of the United Nations [19]. As part of the national health sector development strategy, the Ethiopian Ministry of Health with support from the Clinton Health Access Initiative (CHAI) implemented the EMRI from 2008–2011 with the goal of developing a successful model of rural primary health care that was scalable across the country. This systems-based intervention targeted all patients, not only those with specific diseases, and focused on improving primary health care units (PHCUs), which were supervised by district (or *woreda*) health offices. At the time of the study, PHCUs served a catchment area of about 40,000 people and included one health center with approximately 10–17 staff and an average of 2 health posts each staffed with 2 health extension workers. Since the study, Ethiopia has increased the number of health centers, which now each serve a catchment area of 15,000–25,000 people and work with an average of 6 health posts per health center, each staffed with 2 health extension workers. The EMRI intervention had the following elements: 1) improving infrastructure of health centers (i.e., water, electricity, physical infrastructure, and equipment), 2) improving the supply chain (e.g., transport of specimens and results follow up), 3) building human resource capacity through health worker training and on-site clinical mentoring, 4) developing a system to improve referrals between health posts and health centers, and 5) mobilizing the community with health education. Additionally, the EMRI featured new services including HIV testing at the health posts and establishment of prevention of mother-to-child transmission (PMTCT) of HIV programs at health centers. The EMRI was aligned with the overall health sector strategy of Ethiopia.

### Study design and sample

We conducted an in-depth qualitative study of 7 PHCUs, drawn from a longitudinal study of 20 PHCUs in the EMRI program to generate hypotheses about factors that may influence PHCU performance. We used a positive deviance framework [20], which sought to examine why units within the same context are able to achieve higher performance while other units do not. Using monthly data (collected as part of the monitoring and evaluation of the EMRI program) on 3 targeted outcomes for which Ethiopia had set national performance targets (antenatal care utilization rates, skilled birth attendance rates, and HIV testing rates in antenatal care), we selected 7 PHCUs to undergo further examination using site visits and in-depth qualitative interviews. In each PHCU, we examined both the health center and the health posts, which separate entities but work together to provide primary health care services to their catchment areas. Performance was characterized using 9 months of data, collected from approximately July 2009 to March 2010. We characterized PHCU performance as consistently higher, most improved, or consistently lower.

### Measurement of PHCU performance

Consistently higher performing PHCUs (n = 2) were those that reached or exceeded the EMRI targets for the greatest number of months. The 2 top performing PHCUs met or exceeded the EMRI targets for 48% and 44% of the months, respectively.

Most improved PHCUs (n = 3) were those that met or exceeded 2 of the 3 national targets by the end of the 9-month period and had the steepest rate of improvement during the year. None of the PHCUs met or exceeded the national target for skilled birth attendance rates. To estimate the rate of improvement for each performance indicators, we estimated a regression with the outcome being the performance indicator and the independent variable being time in months. We then arranged the slopes of the regression lines in order from largest to smallest and assigned each slope a rank between 1 and 20. A rank of 1 indicated the steepest regression line and suggested that a health center made the greatest improvement from baseline for that particular indicator. A rank of 20 indicated the lowest slope, or least amount of improvement over time for that PHCU an indicator. The ranks assigned to each PHCU for each of the 3 indicators were summed, creating a cumulative measure of each PHCU improvement over all 3 indicators during the 9 months of the study. Based on these rankings, we selected the health centers with the 1^st^, 2^nd^, and 3^th^ lowest cumulative rankings as the most improved PHCUs.

Consistently lower performing PHCUs (n = 2) were those that met or exceeded national performance targets for the fewest number of months. The two lower performing PHCUs met or exceeded national targets for 15% and 11% of the months, respectively. These health centers also demonstrate a low level of improvement based on the fitted slopes of the performance time trend, ranking 19^th^ and 20^th^ in improvement over the most recent 9 months.

### Data collection

Using standardized data collection methods and staff supervised by the research team, we collected quantitative PHCU performance data included ANC utilization rates, skilled birth attendance rates, and HIV testing rates in antenatal care. ANC utilization rates were calculated as the number of women who initiated ANC divided by the expected number of pregnancies in the catchment area, based on national epidemiologic data. Skilled birth attendance rates were calculated as the number of women who gave birth in the health center divided by the expected number of births in the catchment area, based on data from the Health Management Information System (HMIS) and national census data. HIV testing rates among women in antenatal care was calculated as the number of HIV tests provided to pregnant women at health center or health posts divided by the number of women seen in their first ANC visit. A sample of the reported data was checked and verified with primary records at the PHCU site quarterly by members of the research team.

Qualitative data were attained through site visits and in-depth interviews with key personnel at the health center, the *woreda* health office, and the health posts. At the health center and the *woreda* health office, we sought to interview individuals performing diverse and key roles, including the PHCU coordinator, ANC nurse, health center director, clinical mentor, health extension worker supervisor, and *woreda* health official. At the health post, we conducted interviews of 2 to 3 individuals together with health extension workers and voluntary community health promoters that were available on the days of the site visits. All interviews were guided by a standard discussion guide tailored for different job categories (**Appendix S1**) and conducted in Amharic. Questions included staff roles, challenges that existed and how they were addressed, and perspectives on the quality of services provided to patients and the community. Interviews were conducted by trained research assistants who were fluent in Amharic and supervised by the project manager. The Institutional Review Board at the Yale School of Medicine approved a verbal informed consent process; additionally, they determined that this project presented a minimal risk to participants and waived the requirement to document consent.

Approximately 6 to 8 interviews in total were conducted in each PHCU for a total of 51 interviews. Interviews ranged from 30 to 60 minutes and were audio taped and professionally transcribed to ensure objectivity and reliability of the transcription from Amharic to English.

Site visits observations were two days in length for each PHCU focusing in 4 key domains: health center infrastructure, key management systems (i.e., patient flow, supply chain, pharmacy management, laboratory, supervision and relationships among staff), available services, and general observations. The visits involved meeting with the health center director to discuss the types of services offered by the health center and health post. Site visits also included a tour of the facility where the observer used a structured observation form regarding how care was provided and the patient flow within each department. Health center staff members in each department were interviewed about their roles in the health center and the common challenges they faced in the health center.

### Data analysis

To characterize PHCUs as consistently higher performing, most improved, or consistently lower performing, we used linear regression to estimate the time trends for each outcome indicator and each PHCU; in sensitivity analysis, we experimented with non-linear regression using time-squared and time-cubed in regression models; however, these higher-order terms were non-significant and were therefore dropped from the models.

To determine key themes from the qualitative data, we employed the constant comparison method of qualitative data analysis [21]–[23]. Four members of the research team (RA, TW, JT, EHB) conducted a line-by-line review of transcripts and developed codes inductively. Throughout the coding process, we constantly compared the content with previously coded data to ensure consistent assignment of codes. This iterative process of refining codes including combining codes of like concepts and expanding the properties of each coded concept continued until no new concepts emerged and the final coding structure of 14 codes were established. Using a refined final version of the code structure, two members of the research team (JT, EHB) coded all interview transcripts and resolved disagreements through negotiated consensus.

Once agreement was reached on coding for all PHCUs, we conducted an explicit comparative analysis [21], [24] in which we compared the content of coded material between the 3 performance groups (consistently higher performance, most improved, and consistently lower performance). Results of this comparative analysis formed the basis of the reported results. We used ATLAS.ti (Version 5.0.67; Scientific Software Development GmbH, Berlin, Germany) to facilitate data coding, organization, and retrieval.

## Results

### Overview

A total of 6 themes emerged among the 3 types of PHCUs (consistently higher performing, most improved, and consistently lower performing). These themes included: 1) problem solving capacity at the facility level, 2) relationships between the facilities and the *woreda* (local government) health office, 3) community engagement with health issues, 4) geographical terrain and distance to health centers and health posts, 5) financial budgets for the health centers, and 6) cultural norms regarding use of health services. Although we found similar challenges across all 7 PHCUs in their reports of rough geographic terrain and long distances to services, inadequate financial budgets, and cultural norms that made individuals hesitant to use formal health services, differences across the PHCUs in the themes related to problem solving at the facility level, relationships with the *woreda* health office, and community engagement with health issues were prominent. **Table 1** summarizes these themes and highlights the 3 themes that were distinguished across PHCUs and formed the basis of our hypotheses about factors that may influence PHCU performance.

**Table 1:** Summary of Key Themes.

Theme	Higher performing and most improved PHCUs	Lower performing PHCUs
Problem solving capacity at facility level	Effective staff supervision and management; staff expressed optimism that challenges could be addressed; multiple work flow redesign projects underway	Lack of supervision and management; staff expressed helplessness and lack of control to improve work; recurrent challenges with work processes
Relationships between facilities and government health office	Strong, reliable, and supportive; government health office provided help with transportation, staffing, and supplies; health officer made regular visits and was viewed as cooperative	Distant, non-supportive, and problematic; government health office did not provide transportation or assistance in problem solving; health officers made few visits and were viewed as unhelpful
Community engagement with health issues	Priests, sheiks, women's associations, youth groups, and local government leadership forums helping mobilize efforts to promote use of health centers or health posts	Lack of engagement of religious leadership; limited connections with community agencies or local governmental partners to promote use of health centers or health posts
Geographical terrain and distance to health centers and health posts	Rainy season mud, lack of asphalt roads, long, treacherous distances between homes and facilities	Rainy season mud, lack of asphalt roads, long, treacherous distances between homes and facilities
Financial budgets	Insufficient pay, shortages of necessary medications, poor physical infrastructure	Insufficient pay, shortages of necessary medications, poor physical infrastructure
Cultural norms regarding use of health services	Preference for traditional birth attendants and privacy; focus on farming and income; lack of priority placed on health behaviors	Preference for traditional birth attendants and privacy; focus on farming and income; lack of priority placed on health behaviors

### Distinguishing themes across PHCUs

#### Problem solving within the PHCU

PHCUs that were consistently higher performing and those that were most improved both demonstrated supervision and management that facilitated problem solving, whereas PHCUs that were consistently lower performing had less clear supervision and management of resources, and limited evidence of problem solving. Critical to this theme was the optimism expressed by PHCU staff in the consistently higher performing and most improved PHCUs that challenges would be addressed. In contrast, staff in the consistently lower performing PHCUs expressed feelings of helplessness and lack of control, believing there was nothing they could do to improve PHCU performance. In consistently higher performing PHCUs, staff worked together to redesign the patient flow in order to reduce waiting time and smooth processes whereas inefficient work processes were unaddressed in consistently lower performing PHCUs

The time that patients have to spend waiting has become significantly shorter and they receive quick services. We have reformed our systems. For instance, patients had to go to the cashier's room and return to the card room again and wait in line before they could see a doctor. But now, they would come to the card room and they would straight to OPD after that… Patients [do not] have to waste much time here; they are now able to receive prompt services and return home (#32, clinical mentor, consistently higher performing PHCU).

In addition, staff in higher performing and most improved PHCUs expressed confidence that they could make other changes to improve access to and quality of PHCU services:

We try to manage using what we have at hand… we used to have difficulty about finance for fuel and maintenance for the motorcycle [to reach the health posts]…. We…convinced the people about the objective of the [project] and its sustainability. They understood…that we work for the people…. After they are convinced, they gave…the motorcycle to us (#18, PHCU coordinator, consistently higher performing PHCU).

In contrast, staff in the consistently lower performing PHCUs expressed futility regarding recurrent challenges they faced:

Although we talk with [the PHCU supervisor] all about the problem, there is nothing the supervisor can do because you can go as high as the regional administration but there is nobody that will listen to you (#23, health extension worker, consistently lower performing PHCU).

If I am not able to get gloves, I just work with bare hands without gloves…it [makes me susceptible to infection] but there's nothing I can do (#35, antenatal care nurse, consistently lower performing PHCU).

[When we run out of HIV testing kits] I would be at the health bureau. I would ask them as well at the health center. I would ask these and if there are still no kits, I would just leave it and I do not make calls anywhere else (#37, health extension worker supervisor, consistently lower performing PHCU).

In the consistently higher performing and most improved PHCUs, the role of health care financing reform was viewed as critical to their improvement efforts because it allowed them to retain surplus revenue to be used for approved improvement projects, including reorganizing examination rooms, buying equipment, or keeping essential medications in stock, as described by staff:

Health care financing [reform lets us] make necessary purchases using the permitted budget and do what we can to [serve] the community. For instance, this Xerox machine was purchased through healthcare financing; the patient waiting room was also renovated (#29, health center director, consistently higher performing PHCU).

In one of the most improved PHCUs, they had started a farm to generate revenue for improvements to the health center as described here:

We are currently using the revenue that is obtained from the farm to improve the health center. The money can be used to provide clean running water, pharmaceutical drugs, and the like. If you purchase the medicine with the budget the government provides, the quality of the drugs may not be up to the standard and need of the health center. So by using our own standards and our own money from the farm, the health center can buy different medications that are needed by patients (#9, health center director, most improved PHCU).

These perspectives contrast markedly with the experiences of staff in consistently lower performing PHCUs, who generally reported that they had no options to increase resources. As one PHCU director reported:

We have a lot of rooms but we cannot afford to renovate them. And we also don't have any janitors and we do the cleaning ourselves…. Pens are not supplied on time…and we also have staff shortages. The budget allocated by the *woreda* is not enough and there are now going to be 5 health centers in the *woreda* and when that happens, the problems is going to get even worse (#22, health center director, consistently lower performing PHCU).

#### Relationship with the *woreda* health office

At the consistently higher performing PHCUs, the relationships with the *woreda* health official were strong, reliable, and supportive. In these PHCUs, staff viewed the *woreda* health official as someone who was accessible and helped the PHCU solve problems. For instance, health center directors and a health extension worker supervisor said:

There is a good working relation between the *woreda* and the health center administration. They often come here to visit and undertake supervision. They also give us some assistance…if there is anything that we need from the *woreda*, we don't necessarily wait for the supervision time. We go there in the middle and get whatever we need (#3, health center director, consistently higher performing PHCU).

We get huge assistance from the *woreda*. They supervise every week…by mobile phone and by presenting themselves at the health center. There are annual, quarterly, weekly action plans. They follow up on the implementation of these activities. There are experts assigned to provide support for us (#4, health extension worker supervisor, consistently higher performing PHCU).

In contrast, the experience of the most improved health centers was more mixed; some directors explained that previously the health official had not visited more than once per year and had not helped the health center with its needs. Nevertheless, staff described the *woreda* health officer relationships as improving and becoming more cooperative. For instance, one director said:

Although the support and assistance we are getting from the health office is not satisfactory, they have a mandate…to assess the work that has been done and point out gaps that need further improvement in the health center…frankly speaking, time supervision is lacking, and this can be said in obtaining assistance in other areas, but we are working cooperatively to improve the situation (#9, health center director, most improved PHCU).

In the consistently lower performing PHCUs, the relationship with the *woreda* health official was much more distant and in some cases problematic. Meetings were infrequent and sporadic. As one staff member said:

The *woreda* is 12 kilometers away, so we only go there when the need arises. We do not always go there and we go only there when there are times we have to speak with the *woreda* health officers (#25, PHCU coordinator, consistently lower performing PHCU).

In the consistently lower performing PHCUs, the staff reported that they did not have strong working relationships with the *woreda* health officials and that supervision visits were rare. In these PHCUs, *woreda* health officers were not known to be helpful or provide assistance in solving problems, as expressed by staff:

The first thing that people from the *woreda* and the health center ask us when they come here is ‘how many babies did you deliver?’ But there might be bleedings, and we don't even have gloves here. We can't even get any gloves when we go and ask for them… We are always asking and we are saying that we are missing these things… They do not even supply gloves. We always raise the problem, and the *woreda* always skip it (#23, health extension worker, consistently lower performing PHCU).

#### Community engagement

A major distinction in the consistently higher performing PHCUs was the engagement of community leaders and groups in efforts to mobilize the community to use the health center. Staff described the involvement of priests and sheiks, women's associations, youth groups, the farmer's association, and kebele administration officials in sponsoring health education meetings and encouraging families to listen to the HEWs.

There are priests and there are also sheiks. These people are community leaders; therefore we go to them and we tell them that such and such person is not willing to listen to us and we ask them to help us get through to them. After that, they would go to the community with us and they would tell people that what we had taught them was true; that they should use this education to protect themselves from the virus and to save themselves… They told them they should listen to us (#33, volunteer community health promoter, consistently higher performing PHCU).

Staff in the most improved PHCUs also highlighted the importance of religious elders and government officials; however, the full engagement was less clear. For instance, in some communities, staff described cultural norms that were difficult to change. An HEW in one of these communities said:

The major problem that needs to be addressed urgently is the culture. We could say that there are problems of transportation and finance if the service was provided to the riches and the poor were left out. But the problem is the same for both. There are religious elders and government administrators who are respected among the community. The wives of these persons do deliver in their own houses. If they do not achieve behavioral change, the local people will. (#17, health extension worker, most improved PHCU)

On the other hand, staff in the most improved PHCUs described that religious leaders were successful in influencing people's views and health behaviors in some areas.

There was even one priest who was telling the community that getting tested for HIV does not have anything to do with adultery…and people are starting to ask to be tested themselves. (#47, health extension worker, most improved PHCU).

In the consistently lower performing PHCUs, discussion of linking with religious and government leaders for health was limited or nonexistent. These communities used a man and woman from the neighborhoods to enable the work of the HEWs. For instance, selected neighborhood people would introduce the HEWs, help restrain dogs that might attack HEWs, and provide directions for the HEWs as needed. Nevertheless, HEWs and other PHCU staff did not mention connections with leaders – religious or otherwise – to facilitate their efforts.

### Themes that did not differ markedly across PHCUs

#### Terrain and long distances

Staff in all PHCUs described the challenging terrain of Ethiopia. The challenges, particularly acute in the rainy season but persistent throughout the year, included the lack of asphalt roads, the long distances between homes and health posts and between health posts and health centers, and lack of public transportation.

The roads are rugged and filled with cliffs so we worry about them…the roads are very rough and make it very difficult for health extension workers to come here. So they are afraid to travel here. (#26, volunteer community health promoter, consistently lower performing PHCU).

Because of muddy and difficulty topography, the pregnant women in remote areas will not be able to be picked up by the ambulance car from their home. Thus, we have to carry them… This is one of the problems that we have to deal with until the road is constructed (#7, volunteer community health promoter, most improved PHCU)

#### Inadequate budgets

All PHCUs experienced problems they attributed to inadequate financial resources from the *woreda* health office, the part of the district-level government that supervised and financed the PHCUs. The impact of limited budgets were insufficient staffing and high turnover of existing staff due largely to insufficient pay, shortages of necessary medications, and poor physical infrastructure (i.e., walls, rooms, floors, beds). These budget problems were described by staff of all types of PHCUs.

We have notified the *woreda* [of the equipment shortages]. It will not be enough for all if it is procured through the *woreda* budget. The budget allocated to procure drug supply is little (#16, health extension worker supervisor, most improved PHCU).

There is a need for a data clerk and case manager in the ART service to continue. The *woreda* has to intervene to solve this problem, but because of lack of budget, they haven't given us a response yet (#6, PHCU coordinator, consistently higher performing PHCU).

#### Cultural norms regarding using PHCU services

Prominent and consistent across PHCUs were cultural norms that home delivery was the most appropriate way to give birth, and that delivery with skilled birth attendance was not universally desirable. This perspective reflected the commonly reported belief that birthing at home was safe and that only the highest risk women needed facility-based care. Staff also described that many mothers seemed uncomfortable at a health center, desired more privacy during labor and delivery, and had greater trust in the traditional birth attendants over skilled birth attendants. Some staff attributed cultural norms about health to religion and tradition; other staff perceived a lack of focus on health relative to farming and providing income for their families.

Mothers feel uncomfortable about lying on the delivery couch to deliver because they find it to be too exposing and they feel shy. They feel more comfortable delivering at home inside a closed room with the assistance of a traditional birth attendant and other untrained aids such as the pregnant woman's mother or aunt (#22, health center director, consistently lower performing PHCU).

When we go to the villages to talk about health and health related ideas, some people…are unwilling to accept us. In some cases, they are forced to go back seven or eight times to a single household to convince that person about digging a toilet holes or other health practices… Most people are resistant to implement such ideas because they believe constructing a toilet is not high priority compared to farming, providing food and cloth for their families. (# 8, antenatal care nurse, most improved PHCU).

## Discussion

We found several key themes that distinguished consistently higher performing PHCUs from those that had shown the most improvement and those that were consistently lower performing. Based on these findings, we hypothesize that the managerial problem solving capacity of the PHCU director and staff, the quality of the relationship between the PHCU and the *woreda* (district) health office, and engagement of religious and government leaders in community mobilization efforts for health may be essential for better PHCU performance and effective health systems strengthening efforts. We did not find distinguishing differences in PHCU structural characteristics such as distance from the nearest urban area or the quality of the road to the PHCU. Evidence from this study suggests that health system strengthening efforts should devote substantial resources to not only the technical aspects of improving services but also the more subtle prerequisites for community change.

The implication of our findings for policy-makers, practitioners, and researchers is that these aspects of health systems strengthening – building management capacity at the facility level, improving relationships with local government health offices, and working to mobilize community leadership in health – may be central to effective efforts. Whereas many health system strengthening programs focus on individual clinical and public health interventions, the themes that distinguish higher performance pertain to general problem solving capacity, inter-group relationships, and community leadership. Furthermore, with these factors in place, successful performance was apparent even among PHCUs that experienced similar geographic, financial, and cultural challenges noted by less successful PHCUs, suggesting that investment in management capacity at facilities, government relationships, and community leadership in health may be most central to effect health system strengthening efforts.

Central to the findings was the relationship of the PHCU to the *woreda* health office, manifest especially in the interactions between the PHCU director and the health office director. In cases of higher performing PHCUs, health office resources, such as motorbikes, were shared, the health officer was on-site at the PHCU frequently, and budget issues were viewed as joint problems to be solved. In some areas, PHCUs were allowed to retain some budget to spend more flexibly on PHCU improvements. The finding highlights the importance of full system strengthening efforts, rather than focusing only on the PHCU itself; the government offices that support the PHCU must be integrated into strengthening programs for the program to have maximal impact.

In addition to the relationship with the *woreda* health office, the managerial capacity of the PHCU director and staff, including the HEWs, distinguished the PHCUs. Staff in all PHCUs described substantial challenges and problems within the PHCU including lack of supplies and medications, poor building structures, staff turnover and limited training, and inadequate capital budgets. Nevertheless, in higher performing PHCUs, staff also described multiple instances of problems solving, including creating pleasant coffee ceremonies for families of patients that delivered at the PHCU, reorganizing the flow in the PHCU to reduce crowding, working to renovate and purchase new equipment for the building, and providing data feedback and supervision of staff concerning their performance. Because most health center directors had little managerial training, such skills were not consistent; however, in areas where these skills and behaviors were apparent, performance as measured in this study was also better.

Last, community leadership in health cannot be underestimated. Although multiple staff described Ethiopian cultural norms that did not promote use of formal health services, in some communities, shifts in this culture were possible. These communities were marked by strong leaders in official positions such as priests, sheiks, and government administrators, who were supportive of health improvements. The ability of the PHCUs, especially HEWs, to link with these leaders and use the leaders to advocate for health and facilitate the HEW health messaging was described as critical to improve PHCU utilization. Importantly, religion was viewed as a potentially either negative or positive in its influence on health behavior. Religious beliefs were viewed by some as contributing to beliefs that prenatal care and birthing practices should be private and to the stigmatizing of those who sought HIV testing; at the same time, religious leaders were described as critical to changing people's views and attitudes toward prenatal care, delivery, and HIV testing. The findings underscore the importance of leveraging the power of religion in efforts to change people's health behavior and strengthen health systems.

Our results should be understood in light of study limitations. First, the study involved relatively few PHCUs. Although many more were followed for performance, the 7 that were selected for the study met criteria for higher performing, most improved, and lower performing and were diverse in their location. These 7 PHCUs were studied intensively using rigorous qualitative methods and provide good support for the hypotheses generated. Nevertheless, replication of this study with larger samples and in other geographies of Ethiopia would enhance the transferability of our findings. In particular, studies in the emerging regions of Ethiopia, which were not included in our sample, might identify different themes. Similarly, the study focused on a single country. Although the challenges of rural health care in other low-income settings may be similar, results concerning culture particularly may differ in other countries. Last, as a qualitative study, this study was not designed to test the statistical significance of the factors that we concluded distinguished the PHCUs by their performance. Quantitative studies are needed to establish statistical associations; nevertheless, this qualitative study suggests several key hypotheses for future inquiry.

In sum, health systems strengthening efforts, while essential for improving health and health care, will require intensive investment in several, less technical interventions. These interventions include strengthening the core relationships between the front-line provider units and the government arm that supports and supervises providers' work, the managerial skills within the provider organizations such as PHCUs, and the availability of community leadership that supports and promotes efforts to improve the health and health care services available in their communities. Future studies should examine the statistical association of these factors with PHCU performance and consider as well the impact on longer-term health outcomes such as maternal and child morbidity and mortality.

## Supporting Information

Appendix S1 **Ethiopia Millennium Rural Initiative – Qualitative assessment.** Discussion guides for interviews with health center, *woreda* health office, and health post staff.
(DOC)
