# Correction: A Systems Approach to Improving Rural Care in Ethiopia

**DOI:** 10.1371/annotation/7faaa943-84f2-48e9-9c7e-e19b5b037716

**Published:** 2012-05-07

**Authors:** Elizabeth H. Bradley, Patrick Byam, Rachelle Alpern, Jennifer W. Thompson, Abraham Zerihun, Yigeremu Abebe, Leslie A. Curry

There was a typographical error in the sixth author's name. The correct spelling is Yigeremu Abebe. 

