# Supplementary material for: A Systems Approach to Improving Rural Care in Ethiopia
Source: PLoS One. 2012 Apr 25;7(4):e35042. doi: 10.1371/journal.pone.0035042 (PMC3338815; doi:10.1371/journal.pone.0035042)
Supplement: Appendix S1 — Ethiopia Millennium Rural Initiative – Qualitative assessment. Discussion guides for interviews with health center, woreda health office, and health post staff. (DOC) [file pone.0035042.s001.doc]

**Appendix A**

**Ethiopia Millennium Rural Initiative – Qualitative assessment**

**Objective: To provide insight into the staff’s perspectives regarding health care services provided at the health centers.**

We will use qualitative methods in this part of the study. Therefore, the discussion guides are designed to elicit major themes and are not meant to estimate prevalence or incidence of any events; the sample is purposeful to reflect diversity of staff views and experiences, and the data are open-ended to allow for rich and descriptive detail. The sample will be relatively small as is recommended in qualitative studies to enable greater depth in each interview with the goal of saturated on concepts to understand staff perspectives regarding ’ experiences with the health centers. All people interviewed will be asked to complete a demographic data form.

At each health center, conduct the following:

**Health center based interviews:**

1. Primary Health Care Unit (PHCU) coordinator (CHAI employee)
2. HC clinical mentor (CHAI employee)
3. HC Director (MoH employee, whoever it is – nurse, physician, manager)
4. Antenatal care (ANC) nurse (MoH employee)

**Health post based interviews:**

1. Health extension worker supervisor
2. Heath extension worker (HEW) small group (2-3 participants)
3. Voluntary community health worker (VCHWs) small group (2-3 participants)

**Woreda based interviews:**

1. Woreda health official

**Observation visits:**

1. You will stay for 1-2 days at each HC to observe the practices, how the work is done, and who is doing what to understand what the major problems are, as viewed by the staff and patients. After each visit as soon as possible, debrief with a diary and write up your notes from the day about what the experience has been like.

**HC coordinator, HC clinical mentor, and HC Director one-on-one discussion guides:**

Interviewer’s name:

Date: (M) /(D) /(Y)

Health Center:

Woreda:

**Introduction:**

Thank you for coming to talk with us today. My name is _______, and I work with Yale University, a university based in the USA. We are trying to understand how to make the healthcare provided by your community’s health center and health posts better. Our work is being paid for by the Children’s Investment Fund Foundation, a children’s charity based in England.

We want to hear about your experience delivering healthcare in your community, and your views on the services provided. We’d like to hear what you think is good, what is bad, and what can be made better. There are no right or wrong answers.

Today’s discussion will last about an hour. Your participation is voluntary and you can leave at any time you wish. There is no penalty if you decide not to participate.

Please know that everything you say is confidential. Your name and personal information will not be given to anyone (including other healthcare workers and government officials) and will be removed from all documents and communications. Your participation will in no way affect the healthcare you receive, your job, your family, or yourself.

To make it easier to understand everyone we would like to tape record this conversation. We will only tape record if you say that we can and there is no penalty if you prefer not to tape record. If we have your permission to record, the recordings will be typed up and then reviewed by our research team. In the transcript, your name and any identifiers will not appear. Before we start, do we have your permission to record?

If you have any questions, please feel free to ask them now or at any time.

**Questions for discussion:**

1. Tell me about how you fit into what the health center does? What is **your role**?

Probe: How long have you been doing this?

What is it like working here?

1. We are interested in your **maternal care and childbirth** services. Can you tell us about the care provided for women who are pregnant?

Probe: What do you think influences whether mothers come to the HC

for antenatal care (ANC)?

For the birth?

How has clinical mentoring impacted maternal health services?

1. What are some of the parts of MCH care that are not working so well here in the HC (focus on MCH)? Have there been any changes in the last year? If so, how?

Probe: How did those changes get started?

What challenges did you have to work through to make these changes?

Were there any surprises along the way?

1. What is working well in the HC (focus on MCH)? Is this new? If it has changed in the last year, can you tell me how?

Probe: How did those changes get started?

What challenges did you have to work through to make these changes?

Were there any surprises along the way?

1. What other changes have occurred here in the last year?

Probe: How did those changes get started?

What challenges did you have to work through to make these changes?

Were there any surprises along the way?

1. Can you tell us more about the relationship between the health center management and the woreda? Has anything changed in this relationship in the last year?

Probe: How often do you meet face to face?

How is supervision (supportive or otherwise)?

How do you work to solve problems?

1. Can you tell us about the work of Health Extension Workers and voluntary community health workers? How has their work influenced maternal health and childbirth services during the EMRI program?

Probe: How are they accepted in the community?

What problems do they face? When a problem occurs what happens?

How is the supervision of their work?

1. Overall our goal was to understand what is or isn’t working well at your Health Center. Is there anything else I should have asked you to understand these issues better?

Thanks.

**Discussion guides for the HEW supervisor**

Interviewer’s name:

Date: (M) /(D) /(Y)

Health Center:

Woreda:

**Introduction:**

Thank you for coming to talk with us today. My name is _______, and I work with Yale University, a university based in the USA. We are trying to understand how to make the healthcare provided by your community’s health center and health posts better. Our work is being paid for by the Children’s Investment Fund Foundation, a children’s charity based in England.

We want to hear about your experience delivering healthcare in your community. We’d like to hear what you think is good, what is bad, and what can be made better. There are no right or wrong answers. We want to have an open discussion about your experiences, ideas, and opinions.

Today’s discussion will last about an hour. Your participation is voluntary and you can leave at any time you wish. There is no penalty if you decide not to participate.

Please know that everything you say is confidential. Your name and personal information will not be given to anyone (including other healthcare workers and government officials) and will be disguised in all documents and communications. Your participation will in no way affect the healthcare you receive, your job, your family, or yourself.

To make it easier to understand everyone we would like to tape record the conversation. We will only tape record if you say that we can and there is no penalty if you prefer not to tape record. If we have your permission to record, the recordings will be typed up and then reviewed by our research team. In the transcript, your name and any identifiers will not appear. Before we start, do we have your permission to record?

A few tips to make our discussion go better:

- Please speak one at a time
- Please respect other people’s privacy by not talking about the comments you hear today with anyone else.

If you have any questions, please feel free to ask them now or at any time.

**Questions for HEW Supervisor discussion:**

1. We are interested in your role in **community outreach** with the goal of having more people use the HC when they need it. Can you tell us about your role?

Probe: How long have you been doing this? What is it like working here?

1. We are interested in your experiences supervising health posts in the community. Would you tell us about how you supervise health extension workers?

Probe: How often do you visit them? How has supervision been working?

1. Would you tell us about the services health extension workers offer related to **maternal care and childbirth**?

Probe: What do you think influences whether mothers use antenatal care (ANC) services? Delivery services?

1. Can you tell us more about the relationship of health extension workers within the community? How do community members view your work?

Probe: What problems do they face? When a problem occurs what

happens? How do problems get addressed?

1. What is not working well here at the HP (focus on MCH community mobilization efforts)? Have these changed in the last year? If so, how?

Probe: How did those changes get started?

What challenges did you have to work through to make these changes?

Were there any surprises along the way?

1. What is working well at the HP (focus on MCH community mobilization efforts)? Has this changed in the last year? If so, how?

Probe: How did those changes get started?

What challenges did you have to work through to make these changes?

Were there any surprises along the way?

1. What other changes have occurred here in the last year?

Probe: How did those changes get started?

What challenges did you have to work through to make these changes?

Were there any surprises along the way?

1. Can you tell us about HIV Counseling and Testing at the health post?

Probe: Can you tell us about the availability of HIV testing kits?

How has that impacted your work? When a problem occurs what happens?

How do problems get addressed? Do you have thoughts about how problems might be addressed more effectively?

About how many people per week do health extension workers counsel and/or care for?

1. Overall our goal was to understand what is or isn’t working well at your Health Post and Health Center. Is there anything else I should have asked you to understand these issues better?

Thanks.

**Discussion guides for Health Extension Workers (HEWs)**

Interviewer’s name:

Date: (M) /(D) /(Y)

Health Center:

Woreda:

**Introduction:**

Thank you for coming to talk with us today. My name is _______, and I work with Yale University, a university based in the USA. We are trying to understand how to make the healthcare provided by your community’s health center and health posts better. Our work is being paid for by the Children’s Investment Fund Foundation, a children’s charity based in England.

We want to hear about your experience delivering healthcare in your community. We’d like to hear what you think is good, what is bad, and what can be made better. There are no right or wrong answers. We want to have an open discussion about your experiences, ideas, and opinions.

Today’s discussion will last about an hour. Your participation is voluntary and you can leave at any time you wish. There is no penalty if you decide not to participate.

Please know that everything you say is confidential. Your name and personal information will not be given to anyone (including other healthcare workers and government officials) and will be disguised in all documents and communications. Your participation will in no way affect the healthcare you receive, your job, your family, or yourself.

To make it easier to understand everyone we would like to tape record the conversation. We will only tape record if you say that we can and there is no penalty if you prefer not to tape record. If we have your permission to record, the recordings will be typed up and then reviewed by our research team. In the transcript, your name and any identifiers will not appear. Before we start, do we have your permission to record?

A few tips to make our discussion go better:

- Please speak one at a time
- Please respect other people’s privacy by not talking about the comments you hear today with anyone else.
- If you have any questions, please feel free to ask them now or at any time.

**Questions for HEW small group interview:**

1. We are interested in your role in **community outreach** with the goal of having more people use the HC when they need it. Can you tell us about your role?

Probe: How long have you been doing this? What is it like working here?

1. We are interested in your experiences working at your health post and in the community. Would you tell us about the service you offer related to **maternal care and childbirth**?

Probe: About how many people per week do you each counsel and/or care for?

1. Can you tell us what you find most effective in getting people to use maternal health services at the HC when they need it?

Probe: What do you think influences whether mothers use antenatal care (ANC) services? Delivery services?

1. Can you tell us more about your relationship with the community? How do community members view the services you provide?

Probe: What problems do you face? When a problem occurs with the community what happens? How do problems get addressed?

1. What are some things that affect the work you do in the community?

Probe: The physical infrastructure of the Health Post? Transportation issues?

Weather? Support from the woreda? Training? Availability of drugs/supplies?

1. Can you tell us about the supervision you receive?

Probe: How often are you visited by the HEW supervisor? How often do you meet face to face?

How is supervision (supportive or otherwise)? How do you solve problems together? Other?

1. Can you tell us about your work with the voluntary community health workers? Has anything changed in this relationship in the last year?

Probe: How often do you meet face to face? What problems do you face? When a problem occurs what happens? How do problems get addressed?

1. Can you tell us about HIV Counseling and Testing at the health post?

Probe: Can you tell us about the availability of HIV testing kits? How has that impacted your work? When a problem occurs what happens?

How do problems get addressed? About how many people per week do you each counsel and/or care for?

1. Overall our goal was to understand what is or isn’t working well at your Health Post and Health Center. Is there anything else I should have asked you to understand these issues better?

Thanks.

**Discussion guide for Voluntary Community Health Workers (VCHWs)**

Interviewer’s name:

Date: (M) /(D) /(Y)

Health Center:

Woreda:

**Introduction:**

Thank you for coming to talk with us today. My name is _______, and I work with Yale University, a university based in the USA. We are trying to understand how to make the healthcare provided by your community’s health center and health posts better. Our work is being paid for by the Children’s Investment Fund Foundation, a children’s charity based in England.

We want to hear about your experience delivering healthcare in your community. We’d like to hear what you think is good, what is bad, and what can be made better. There are no right or wrong answers. We want to have an open discussion about your experiences, ideas, and opinions.

Today’s discussion will last about an hour. Your participation is voluntary and you can leave at any time you wish. There is no penalty if you decide not to participate.

Please know that everything you say is confidential. Your name and personal information will not be given to anyone (including other healthcare workers and government officials) and will be disguised in all documents and communications. Your participation will in no way affect the healthcare you receive, your job, your family, or yourself.

To make it easier to understand everyone we would like to tape record the conversation. We will only tape record if you say that we can and there is no penalty if you prefer not to tape record. If we have your permission to record, the recordings will be typed up and then reviewed by our research team. In the transcript, your name and any identifiers will not appear. Before we start, do we have your permission to record?

A few tips to make our discussion go better:

- Please speak one at a time
- Please respect other people’s privacy by not talking about the comments you hear today with anyone else.
- If you have any questions, please feel free to ask them now or at any time.

**Questions for VCHW small group interview:**

1. We are interested in your role in **community outreach** with the goal of having more people use the HC when they need it. Can you tell us about your role?

Probe: How long have you been doing this? What is it like working here?

1. We are interested in your experiences working at your health post and in the community. Would you tell us about the service you offer related to **maternal care and childbirth**?

Probe: About how many people per week do you each counsel and/or care for?

1. Can you tell us what you find most effective in getting people to use maternal health services at the HC when they need it?

Probe: What do you think influences whether mothers use antenatal care (ANC) services? Delivery services?

1. Can you tell us more about your relationship the community? How do community members view the services you provide?

Probe: What problems do you face? When a problem occurs with the community what happens? How do problems get addressed?

1. What are some things that affect the work you do in the community?

Probe: The physical infrastructure of the Health Post? Transportation issues?

Weather? Support from the woreda? Training? Availability of drugs/supplies?

1. Can you tell us about the supervision you receive?

Probe: How often are you visited your supervisor? How often do you meet face to face?

How is supportive supervision? How do problems get addressed? Other?

1. Can you tell us about your work with health extension workers? Has anything changed in this relationship in the last year?

Probe: How often do you meet face to face? What problems do you face? When a problem occurs what happens? How do problems get addressed?

1. Can you tell us about HIV Counseling and Testing at the health post?

Probe: Can you tell us about the availability of HIV testing kits? How has that impacted your work?

1. Overall our goal was to understand what is or isn’t working well at your Health Post and Health Center. Is there anything else I should have asked you to understand these issues better?

Thanks.

**Woreda official one-on-one discussion guide:**

Interviewer’s name:

Date: (M) /(D) /(Y)

Health Center:

Woreda:

**Introduction:**

Thank you for coming to talk with us today. My name is _______, and I work with Yale University, a university based in the USA. We are trying to understand how to make the healthcare provided by your community’s health center and health posts better. Our work is being paid for by the Children’s Investment Fund Foundation, a children’s charity based in England.

We want to hear about your experience delivering healthcare in your community, and your views on the services provided. We’d like to hear what you think is good, what is bad, and what can be made better. There are no right or wrong answers.

Today’s discussion will last about an hour. Your participation is voluntary and you can leave at any time you wish. There is no penalty if you decide not to participate.

Please know that everything you say is confidential. Your name and personal information will not be given to anyone (including other healthcare workers and other government officials) and will be removed from all documents and communications. Your participation will in no way affect the healthcare you receive, your job, your family, or yourself.

To make it easier to understand everyone we would like to tape record this conversation. We will only tape record if you say that we can and there is no penalty if you prefer not to tape record. If we have your permission to record, the recordings will be typed up and then reviewed by our research team. In the transcript, your name and any identifiers will not appear. Before we start, do we have your permission to record?

If you have any questions, please feel free to ask them now or at any time.

**Questions for discussion:**

1. Tell me about your role in this woreda?

Probe: How long have you been doing this? What is it like working here?

1. Can you tell us about the _______ health center that the Clinton Foundation is working with for the Ethiopian Millennium Rural Initiative? How would you describe the performance of this health center?

Probe: What is working well at that HC (focus on MCH)? Is this new? If it has changed in the last year, can you tell me how?

What challenges did you have to work through to make these changes?

Were there any surprises along the way?

1. Now please think about comparable health centers where the Clinton Foundation EMRI is not operating. How do they perform relative to EMRI health centers? What is the same? What is different?
2. What do you believe have been the major contributions of the Clinton Foundation work in health centers and health posts in your area? What has not gone so well with this work?
3. Can you tell us more about your relationship with the health center management? Has anything changed in this relationship in the last year?

Probe: How often do you meet face to face? And how do you communicate in between meetings?

What is the supervision like (supportive or otherwise)?

How do you work to solve problems?

6. Overall our goal was to understand what is or isn’t working well at your Health Center. Is there anything else I should have asked you to understand these issues better?

Thanks.
